# Supplementary material for: MetaRibo-Seq measures translation in microbiomes
Source: Nat Commun. 2020 Jun 29;11:3268. doi: 10.1038/s41467-020-17081-z (PMC7324362; doi:10.1038/s41467-020-17081-z)
Supplement: Supplementary file 10 — Supplementary Data 7 [file 41467_2020_17081_MOESM10_ESM.zip › File2/Confidence_High_Taxonomy/48650_out.krona.html]

Javascript must be enabled to view this page.

members
magnitude
magnitudeUnassigned
count
unassigned
taxon
rank

48650\_out

2

superkingdom
1
2

1239
1
phylum

class
1
91061

1385
1
order

family
1
186822

species

SRS148159\_contig\_number\_2560
2003592
1


SRS104311\_contig\_number\_contig-100\_16156.157769
1
